# Stepwise approach for combining many sources of evidence for site-recognition in genomic sequences

**DOI:** 10.1186/s12859-016-0968-y

**Published:** 2016-03-05

**Authors:** Javier Pérez-Rodríguez, Nicolás García-Pedrajas

**Affiliations:** Department of Computing and Numerical Analysis, University of Córdoba, Córdoba, 14071 Campus de Rabanales Spain

**Keywords:** Site recognition, Combination of evidence, Translation initiation site recognition, Stop codon recognition

## Abstract

**Background:**

Recognizing the different functional parts of genes, such as promoters, translation initiation sites, donors, acceptors and stop codons, is a fundamental task of many current studies in Bioinformatics. Currently, the most successful methods use powerful classifiers, such as support vector machines with various string kernels. However, with the rapid evolution of our ability to collect genomic information, it has been shown that combining many sources of evidence is fundamental to the success of any recognition task. With the advent of next-generation sequencing, the number of available genomes is increasing very rapidly. Thus, methods for making use of such large amounts of information are needed.

**Results:**

In this paper, we present a methodology for combining tens or even hundreds of different classifiers for an improved performance. Our approach can include almost a limitless number of sources of evidence. We can use the evidence for the prediction of sites in a certain species, such as human, or other species as needed. This approach can be used for any of the functional recognition tasks cited above. However, to provide the necessary focus, we have tested our approach in two functional recognition tasks: translation initiation site and stop codon recognition. We have used the entire human genome as a target and another 20 species as sources of evidence and tested our method on five different human chromosomes. The proposed method achieves better accuracy than the best state-of-the-art method both in terms of the geometric mean of the specificity and sensitivity and the area under the receiver operating characteristic and precision recall curves. Furthermore, our approach shows a more principled way for selecting the best genomes to be combined for a given recognition task.

**Conclusions:**

Our approach has proven to be a powerful tool for improving the performance of functional site recognition, and it is a useful method for combining many sources of evidence for any recognition task in Bioinformatics. The results also show that the common approach of heuristically choosing the species to be used as source of evidence can be improved because the best combinations of genomes for recognition were those not usually selected. Although the experiments were performed for translation initiation site and stop codon recognition, any other recognition task may benefit from our methodology.

## Background

The recognition of functional sites within the genome is one of the most important problems in Bioinformatics research. Determining where different functional sites, such as the promoters, translation start sites, translation initiation sites (TISs), donors, acceptors and stop codons, are located provides useful information for many tasks. For instance, the recognition of translation initiation sites, donor, acceptors and stop codons [1] is a basic in any program developed to perform a gene recognition task. Most current gene structure prediction programs start with a site recognition step [2] and, once putative sites have been discovered, they try to combine them into meaningful gene structures. It is evident that this site recognition step is crucial as, in most cases, if the sites for a gene are not identified that gene will no longer be considered by the program. On the other hand, if many false positives are detected it is likely that the gene recognition program performance would be seriously damaged. State-of-the-art site recognizers use complex classifiers, namely support vector machines (SVMs), and medium upstream and downstream sequences from the putative sites [1, 3–5].

Recent approaches [2] for human gene recognition also make use of the information available for other species to improve the recognition of the functional sites. However, the combination is carried out in a heuristic way. The species used for comparison are arbitrarily chosen, using the widely assumed hypothesis that we must consider moderately distant evolutionary relatives. Furthermore, the classifiers used for recognition of the sites in each species are also arbitrarily chosen. The best classifiers are usually chosen without considering the relevant topic of classifier diversity [6] which is crucial in any combination of learners [7]. It is unlikely that such a process would produce the best possible result. Due to the large number of available species and the large number of different classifiers that can be applied to make use of such information, a systematic method for obtaining the best possible combination is highly desirable.

In this work, we propose a principled approach in which we can consider as many different sources of evidence as available and use as many different classifiers as needed. A rapid validation process constructs a near-optimal combination that achieves a better performance than any of its members. To obtain a method that can be scaled up to as many sources of information as needed, we use a greedy stepwise approach. Two alternatives are designed, one based in a constructive approach beginning with an empty model and another based on a destructive approach beginning with a model considering all available sources of evidence. Then, a stepwise procedure is applied until no further improvement is observed in the obtained model. From the point of view of Machine Learning, these two approaches are usually named as forward selection and backward elimination respectively.

## Methods

Our aim is to develop a methodology for combining tens or even hundreds of classifiers for site recognition. From a machine learning perspective, such a problem is usually approached differently depending on the computational cost of the available solutions. The optimum approach is the exhaustive evaluation of all possible combinations of classifiers. However, if we have *N* trained classifiers, the number of possible combinations is 2^*N*^−1, which is prohibitive even for moderate values of *N*. Thus, we must resort to optimization algorithms that will perform a guided search in the space of possible solutions. For the problem of finding the optimal solution, any of the many metaheuristics available in the machine learning literature, such as evolutionary computation [8], particle swarm optimization [9], ant colonies [10] or differential evolution [11], could be used. However, all of these methodologies require the repetitive evaluation of many solutions to achieve their optimization goal. In the problem of site recognition, the evaluation of a possible solution is a costly process due to the large datasets involved. Thus, these metaheuristics are not feasible.

To avoid the computational cost of these metaheuristics, we developed a different approach. We used a stepwise greedy approach in both a constructive and a destructive way, which requires evaluating significantly fewer solutions. The process for obtaining the best combination of classifiers for different species is composed of two main stages: training stage and validation stage. Before starting the learning process, we need the training datasets, the testing dataset and the validation dataset. Without loss of generality and to provide the necessary focus for our description, we will use here the same setup of the reported experiments below. We will address the problem of site recognition in the human genome. To solve this problem, we will use as a test set the sites of a certain chromosome, *C*. The training set will be all the remaining human chromosomes and the genomes of all the species we want. As validation, we will chose one of the human chromosomes in the training set, *V*, and remove it from the training set.

For the training stage, we select as many species as could be useful for our problem. We need not select the most appropriate ones because the stepwise validation stage will discard the useless classifiers. Once we have selected the set of species whose genomes we are going to use, we train as many classifiers as we want from those species. For every organism, we can train different classifiers, such as support vector machines (SVMs), neural networks (NNs), decision trees (DTs), the *k*-Nearest Neighbor (*k*-NN) rule or the same classifier methods with different parameters. Because the validation stage can consider hundreds of classifiers, any method of potential interest can be used. Again, the validation stage will remove unneeded classifiers.

Once we have the trained classifiers, we will perform the validation stage, whose aim is to obtain the best possible combination of classifiers. For that purpose, we designed two different approaches. Both of these approaches are stepwise greedy approaches. We developed a constructive incremental approach and a destructive decremental approach. In the incremental approach, we begin by evaluating all the classifiers in the validation set *V*. The best one, *c*_1_, is added to the set of selected classifiers, which was empty. Then, the evaluation is conducted again using *c*_1_ together with all the remaining classifiers. The best combination is chosen, and a second classifier, *c*_2_, is added. The process is repeated until the addition of a new classifier does not improve the validation accuracy. The constructive method is depicted in Algorithm 1.



For the destructive approach, we start with a model with all the available classifiers, *n*, {*c*_1_,*c*_2_,…,*c*_*n*_}. One by one, every classifier is removed from the set, and the set is reevaluated using the validation set. If all of the classifiers have a positive effect on the validation accuracy, the process is stopped. Otherwise, the worst performing classifier is removed and the process is repeated until the stop criterion is met. The destructive method is depicted in Algorithm 2.



Another issue must be considered for our approach. We must determine how the different classifiers are combined. In the machine learning literature, combining different sources of evidence for a classification problem is a common task [12]. Although various sophisticated methods have been developed for combining many classifiers [13–16], in a practical sense, none of them are able to beat the simpler methods on a regular basis. Thus, we have considered three commonly used simple methods to combine the classifiers: sum of outputs, majority voting and maximum output. These methods are fairly straightforward. The combination using the sum of outputs simply adds together the outputs of all the models. The majority voting scheme counts the classification given by every model and outputs the most common case. The maximum approach uses only the classifier whose output has the highest absolute value.

For these three methods to be useful, we must consider the different ranges of their outputs and the different optimal decision thresholds of the five classification method we will use. To account for the different ranges, all the outputs of the methods were scaled to the interval [−1,1]. To account for the different thresholds, we obtain the optimal threshold for each method, *th*_*otpimal*_, by cross-validation and we obtain the effective output of every classifier, which is given by *y*−*th*_*opimal*_, where *y* is the actual output of the classifier.

With the three combination methods and the two stepwise algorithms, we have for any performance measure selected six different combinations of models. For any recognition task and any performance measure, we will obtain these six models and return as a final result of our methodology the best combination in terms of cross-validation performance.

### Experimental setup

To test our model, we chose the human genome together with other 20 species. Our aim was to test whether any species, regardless of its closeness with the human genome, could be useful. The following species were considered:^1^ Anolis carolinensis (AC), Bos primigenius taurus (BT), Caenorhabditis elegans (CE), Callithrix jacchus (CJ), Canis lupus familiaris (CLF), Danio rerio (DR), Drosophila melanogaster (DM), Equus caballus (EC), Ficedula albicollis (FA), Gallus gallus (GG), Homo sapiens (HS), Macaca mulatta (MaM), Monodelphis domestica (MD), Mus musculus (MM), Ornithorhynchus anatinus (OA), Oryctolagus cuniculus (OC), Pan troglodytes (PT), Rattus norvegicus (RN), Schistosoma mansoni (SM), Sus scrofa (SS) and Takifugu rubripes (TR). These genomes were selected to have a wide variety of organisms whose genomes are fully annotated.

We also used annotated mRNA sequences of Bos taurus (BT.RNA), Danio rerio (DR.RNA), Homo sapiens (HS.RNA), Mus musculus (MM.RNA), Rattus norvegicus (RN.RNA), Sus scrofa (SS.RNA) and Xenopus tropicalis (XT.RNA). Such sequences were screened from RefSeq mRNA curated records downloaded from NCBI RefSeq ftp (ftp://ftp.ncbi.nlm.nih.gov/refseq/) (Last updated: November 17, 2014). The species-specific RefSeq directories provide a cumulative set of records for transcripts and proteins for those species. Records with no annotation for start or stop codons were eliminated. For every training set, regardless of the species, we removed the genes that were shared with the test chromosome for all the training datasets.

Five classifiers were trained from every dataset: the stop codon method [17], a decision tree, a *k*-nearest neighbor rule, a positional weight matrix and a support vector machine with a string kernel. The parameters for every classifier were obtained using 10-fold cross-validation. For learning the classification models we used random-undersampling, for validation and testing the datasets were used unmodified. Thus, a total of 140 models were trained for every dataset.

Another key parameter of the learning process is the window around the functional site that is used to train the classifiers. The value of the window for each classifier was obtained by cross-validation. We tested the performance of the following windows: [−100,0], [−75,25], [−50,0], [−50,50], [−25,0], [−25,25], [−25,75], [−10,15], [−10,40], [−10,90], [0,25], [0,50] and [0,100]. For each trained classifier, the best window was chosen. For the stop codon method, we used the additional window values of [0,200], [0,300], [0,400] and [0,500] for TIS recognition and the window values of [−200,0], [−300,0], [−400,0] and [−500,0] for stop codon recognition.

Our approach was evaluated using human chromosomes 1, 3, 13, 19 and 21 for testing and human chromosome 16 for validation. These datasets are shown in Table 1. We used all TISs and stop codons of the CCDS Update Released for Human of September 7, 2011. This update uses Human NCBI build 37.3 and includes a total of 26,473 CCDS IDs that correspond to 18,471 GeneIDs.

**Table 1:** Random undersampling was used for training; thus, the number of negative instances was equal to the number of positive instances

Dataset		Training data	Testing data	
		Positives/Negatives	Positives	Negatives
Chr. 1	TIS	17,638	2156	8,074,590
	STOP	17,404	2154	23,573,031
Chr. 3	TIS	18,631	1163	7,291,951
	STOP	18,444	1114	21,522,500
Chr. 13	TIS	19,454	340	3,664,164
	STOP	19,225	333	10,878,302
Chr. 19	TIS	18,383	1411	1,698,891
	STOP	18,136	1422	4,665,804
Chr. 21	TIS	19,561	233	1,303,634
	STOP	19,558	237	3,726,959

As SVMs with weighted degree (WD) kernel has consistently proven to be the best state-of-the-art method for site-recognition [5, 18] we chose this method as our baseline approach. To assure a fair comparison, we considered not only these methods but also all others used in classifiers. Then, for every experiment, we compared our approach to the best performing method in terms of validation performance. In fact, SVM with WD kernel was always the best individual classifier. Table 2 summarizes the hyperparameters used to train the classification models.

**Table 2:** Hyperparameters for the different classifiers. For all of them random undersampling [23] was used

Classifier	Inputs	Hyperparameters
Decision trees	Raw sequence	Pruned trees
Position weight matrix	Raw sequence	None
Stop codon method	Raw sequence	None
Support vector	Raw sequence	*C*∈{1,10,100,100},*d*∈{12,24}
machines		
k nearest neighbor	Raw sequence	Hamming distance, *k*∈[1,100]

To evaluate the obtained classifiers, we used the standard measures for imbalanced data. Given the number of true positives (TP), false positives (FP), true negatives (TN) and false negatives (FN), we used the sensitivity, $Sn = \frac {TP}{TP + FN}$, and the specificity, $Sp = \frac {TN}{TN + FP}$. The geometric mean of these two measures, $G-\text {mean} = \sqrt {Sp \cdot Sn}$, will be our first classification metric. As a second measure, we used the area under the receiver operating characteristic (ROC) curve (auROC). However, auROC is independent of class ratios, and it can be less meaningful when we have very unbalanced datasets [5]. In such cases, area under the precision recall curve (auPRC) can be used. This measure is especially relevant if we are mainly interested in the positive class. However, it can be very sensitive to subsampling. In our results, we use all the positive and negative instances for each of the five chromosomes tested, so no subsampling is used. This also yields small auPRC values.

We use these three metrics because they provide two different views of the performance of the classifiers. The auROC and auPRC values describe the general behavior of the classifier. However, when used in practice, we must establish a threshold for the classification of a query pattern. *G*-mean provides the required snapshot of the performance of the classifier when we set the required threshold.

## Results and discussion

As stated, we performed experiments for the recognition of TISs and stop codons to provide the necessary focus. However, our approach is applicable to any recognition task. The experiments had two different objectives. We wanted to know which species were more useful for the recognition of the two functional sites. We challenged the general heuristic method of selecting a species based on biological considerations alone. We also wanted to compare the results using our method with the standard procedure of selecting the best performing model, which is the common approach in the literature. In the following two sections, we discuss the results for TIS and stop codon recognition.

### Results for TIS recognition

One of the advantages of our approach is that we can optimize for the performance measure that we are interested in, which can be the *G*-mean, the auROC, the auPRC or any other measure useful for our application. Thus, we conducted our experiments using three performance measures: *G*-mean, auROC and auPRC. The first relevant result is that the combination of best models obtained for each measure was different. This means that, depending on the aim of the work, different combinations of classifiers are needed.

For each of the five studied chromosomes, we obtained three different combinations of models, each optimized for one of the three measures mentioned above. As a general rule, the constructive method always outperformed the destructive method. The latter always obtained combinations of many more models that exhibited over-fitting and worse performance. It is also interesting to note the homogeneous behavior across the different chromosomes. For all of the five chromosomes, the combination that achieved the best results was the sum for auROC and auPRC and majority for *G*-mean. The combination based on the maximum output was never the best-performing one. In this latter combination method, the effect of a bad classifier was too harmful to obtain good performance. In this paper, for brevity’s sake, only the best models are reported.

Once we established the best stepwise method and the best combination, we examined the results in terms of the species involved in the best combinations. Table 3 shows the models selected for the best combination for each measure and each chromosome. Regardless of the optimized measure, there was only one species that never appeared in the best combination: CE. This result indicates that, although the contribution of certain species is more relevant than others, the information of many genomes was useful for the prediction of human TISs, even those species that are very distant relatives of humans. Another interesting result is the fact that, for the three different measures, auROC, auPRC and *G*-mean, the obtained combinations of models were quite different. This result indicates that we must consider our aims before designing our classifier. In most previous works, that is not taken into account.

**Table 3:** The table shows the models selected for all methods and the five studied chromosomes for TIS recognition

			CE					AC					DM					OA					HS					OC					BT				
Chrom.	Obj.	#	S	C	K	P	W	S	C	K	P	W	S	C	K	P	W	S	C	K	P	W	S	C	K	P	W	S	C	K	P	W	S	C	K	P	W
1	auROC	8																																			
	auPRC	29								X					X					X										X					X		
	G	2																																			
3	auROC	8																																			
	auPRC	33								X					X										X		X			X				X			
	G	2																																			
13	auROC	7													X																						
	auPRC	28								X										X					X					X					X		
	G	2																																			
19	auROC	7																																			
	auPRC	27																												X					X		X
	G	2																																			
21	auROC	8																																			
	auPRC	31								X																				X		X		X	X		
	G	2																																			
			CJ					FA					EC					CLF					GG					MD					SM				
Chrom.	Obj.	#	S	C	K	P	W	S	C	K	P	W	S	C	K	P	W	S	C	K	P	W	S	C	K	P	W	S	C	K	P	W	S	C	K	P	W
1	auROC	8					X													X																	
	auPRC	29			X					X			X							X					X					X							
	G	2																																			
3	auROC	8					X													X					X												
	auPRC	33			X		X			X					X					X					X								X				
	G	2																																			
13	auROC	7															X			X																	
	auPRC	28			X		X			X					X					X					X								X				
	G	2																																			
19	auROC	7																		X																	
	auPRC	27					X			X		X	X		X					X					X					X			X				
	G	2																																			
21	auROC	8													X		X								X												
	auPRC	31		X	X		X			X			X		X		X			X					X					X			X				
	G	2																																			
			MM					DR					MaM					TR					PT					SS					RN				
Chrom.	Obj.	#	S	C	K	P	W	S	C	K	P	W	S	C	K	P	W	S	C	K	P	W	S	C	K	P	W	S	C	K	P	W	S	C	K	P	W
1	auROC	8													X		X										X										
	auPRC	29		X	X		X							X	X							X		X	X												X
	G	2															X										X										
3	auROC	8															X										X										
	auPRC	33		X	X					X				X	X					X				X	X		X			X					X		X
	G	2															X										X										
13	auROC	7															X										X			X							
	auPRC	28								X				X	X		X			X		X		X													X
	G	2															X																				
19	auROC	7													X		X										X	X									
	auPRC	27			X		X			X				X						X		X							X	X					X		
	G	2															X										X										
21	auROC	8															X										X										
	auPRC	31					X			X				X						X		X			X										X		X
	G	2															X										X										
			BT.RNA					DR.RNA					HS.RNA					MM.RNA					RN.RNA					SS.RNA					XT.RNA				
Chrom.	Obj.	#	S	C	K	P	W	S	C	K	P	W	S	C	K	P	W	S	C	K	P	W	S	C	K	P	W	S	C	K	P	W	S	C	K	P	W
1	auROC	8		X													X																X				
	auPRC	29			X		X			X					X										X					X		X	X		X		
	G	2																																			
3	auROC	8															X							X									X				
	auPRC	33													X							X		X	X					X		X	X		X		
	G	2																																			
13	auROC	7															X																X				
	auPRC	28			X										X		X								X					X		X	X		X		
	G	2															X																				
19	auROC	7															X											X									
	auPRC	27			X					X					X		X								X					X		X			X		
	G	2																																			
21	auROC	8												X			X																X				
	auPRC	31			X		X			X					X					X									X	X		X			X		
	G	2																																			

S stands for stop codon method, C for C4.5, K for *k*-NN, P for PWM and W for a SVM with a WD string kernel

Regarding the classification models, PWM was never chosen. The stop codon method was chosen for EC and SM. The decision tree trained with the C4.5 algorithm was selected several times, but the *k*-NN rule and the SVM with a string kernel were the most frequently selected methods. The case of *k*-NN is remarkable as it is not usually used for this task [1, 17, 19, 20]. It appears that the diversity that *k*-NN introduced in the models was useful for the overall performance of the combinations, despite of the fact that *k*-NN alone showed worse performance than an SVM alone. In classifier ensembles literature [6] is already stated that classifier diversity is a desired feature for improving the performance of the ensemble. Thus, the diversity introduced by these models might be the reason of their inclusion in the best combination. EC, CLF, MaM and PT were the species most frequently chosen. It is interesting to note that HS was seldom used.

With respect to the three different objectives, optimizing the *G*-mean showed the most stable results. For the five chromosomes, the selected models were always the SVM method for MaM and PT. For auROC, seven or eight models were selected. The SVM method was always chosen for MaM and PT, but the remaining methods and species depended on the chromosome. This is another interesting result because most TIS recognition programs mainly rely on common models for any task. Finally, for auPRC, significantly more models were selected, from 27 to 33, with a significant variation between the chromosomes. Here, the large number of negative samples made this task harder than optimizing the other two criteria.

**Fig. 1:**
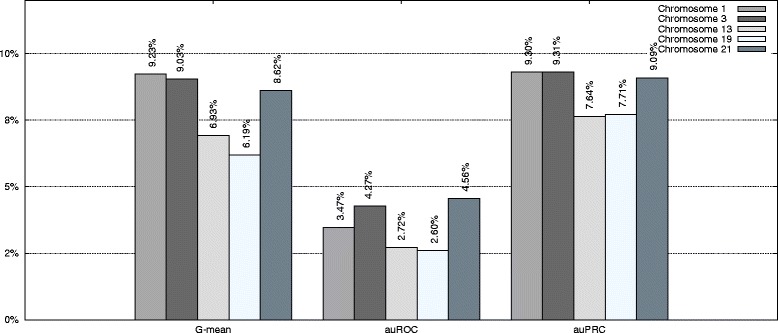
Improvement for TIS recognition. The figure shows the improvement of our approach with respect to the standard approach of using the best performing classifier

The next step was to compare the performances of our approach and the standard method of choosing the best performing classifier. Overall results for TIS recognition problem for the five studied human chromosomes is shown in Table 4. A first conclusion is that the stepwise method was able to improve the standard approach for all three measures and all five chromosomes. The improvements in auROC, auPRC and *G*-mean are shown in Fig. 1. The table also shows the ability of our approach to find the combination in a reasonably short time. In the worst case only 3708 seconds are needed.

**Table 4:** The table shows the specificity (Sp), sensitivity (Sn), true positives (TP), true negatives (TN), false negatives (FN), false positives (FP) and area under the ROC and PRC curves (auROC/PRC) as well as the time needed for obtaining the best combination for all methods and the five studied chromosomes for TIS recognition

Chromosome	Objective	Method	Combination	auROC	auPRC	G	Sp	Sn	TP	FN	TN	FP	#models	Time (s)
Chr. 1	-	Std	-	0.9473	0.0929	0.8430	0.9528	0.7458	1608	548	7,693,177	381,413	-	-
	auROC	Cons	Sum	**0.9820**	0.1529	0.8893	0.9874	0.8010	1727	429	7,972,989	101,601	8	1555
	auPRC	Cons	Sum	0.9669	**0.1859**	0.8326	0.9859	0.7032	1516	640	7,960,941	113,649	29	3436
	G	Cons	Majority	0.9445	0.0074	**0.9353**	0.9599	0.9114	1965	191	7,750,802	323,788	2	572
Chr. 3	-	Std	-	0.9334	0.0831	0.8335	0.9584	0.7248	843	320	6,988,324	303,627	-	-
	auROC	Cons	Sum	**0.9761**	0.1449	0.8458	0.9929	0.7206	838	325	7,240,060	51,891	8	1807
	auPRC	Cons	Sum	0.9596	**0.1762**	0.7891	0.9920	0.6277	730	433	7,233,286	58,665	33	3708
	G	Cons	Majority	0.9315	0.0049	**0.9238**	0.9664	0.8831	1027	136	7,046,822	245,129	2	696
Chr. 13	-	Std	-	0.9506	0.0844	0.8692	0.9479	0.7971	271	69	3,473,147	191,017	-	-
	auROC	Cons	Sum	**0.9778**	0.1347	0.8429	0.9940	0.7147	243	97	3,642,333	21,831	7	1348
	auPRC	Cons	Sum	0.9692	**0.1608**	0.7924	0.9929	0.6324	215	125	3,638,155	26,009	28	3280
	G	Cons	Majority	0.9483	0.0038	**0.9385**	0.9630	0.9147	311	29	3,528,645	135,519	2	476
Chr. 19	-	Std	-	0.9456	0.1277	0.8727	0.9068	0.8398	1185	226	1,540,551	158,340	-	-
	auROC	Cons	Sum	**0.9716**	0.1726	0.8876	0.9691	0.8129	1147	264	1,646,337	52,554	7	1510
	auPRC	Cons	Sum	0.9575	**0.2048**	0.8534	0.9657	0.7541	1064	347	1,640,628	58,263	27	3269
	G	Cons	Majority	0.9436	0.0181	**0.9346**	0.9555	0.9142	1290	121	1,623,271	75,620	2	473
Chr. 21	-	Std	-	0.9353	0.0726	0.8409	0.9415	0.7511	175	58	1,227,429	76,205	-	-
	auROC	Cons	Sum	**0.9809**	0.1299	0.8612	0.9875	0.7511	175	58	1,287,377	16,257	8	1526
	auPRC	Cons	Sum	0.9670	**0.1635**	0.8201	0.9857	0.6824	159	74	1,284,991	18,643	31	3352
	G	Cons	Majority	0.9362	0.0043	**0.9271**	0.9582	0.8970	209	24	1,249,122	54,512	2	288

The results of our method for the three different evaluation measures are shown in boldface

The differences were significant. For *G*-mean, in the worst case, the improvement was 6.19 %, and in the best case, it was 9.23 %. For auPPRC, the results were even better, from 7.64 to 9.31 %. For auROC, the improvement was less significant, but it still ranged from 2.60 to 4.56 %.

Table 5 shows the relative improvement of our approach in terms of the numbers of true positive, false negatives, true negatives and false positives. In the table, we can see how our approach was able to improve the false negative results for the case with least improvement by 46 % and for the case with largest improvement by 65 %. Most of the programs for gene recognition used nowadays include a first step of TIS recognition. From that step the corresponding methods are used to obtain the whole gene structure prediction. A gene whose TIS is missed by this step would be completely ignored by those program. Thus, the proposed method as it improves the TIS recognition accuracy would be able to improve the performance of any of these gene recognition programs.

**Table 5:** Relative improvement for true positives, false negatives, true negatives and false positive of our approach over the best method for TIS recognition

Chromosome	True	False	True	False
	positive	negative	negative	positive
Chr. 1	22.20 %	65.15 %	0.75 %	15.11 %
Chr. 3	21.83 %	57.50 %	0.84 %	19.27 %
Chr. 13	14.76 %	57.97 %	1.60 %	29.05 %
Chr. 19	8.86 %	46.46 %	5.37 %	52.24 %
Chr. 21	19.43 %	58.62 %	1.77 %	28.47 %

Furthermore, our method was also able to improve the false positive rate, from 15 to 52 % depending on the chromosome. This is a significant reduction in the number of putative TIS that are fed to any gene recognition system so a significant improvement in its accuracy might also be expected. This would be especially true when the large amount of false positives found by the standard approach is an actual problem for any automatic annotation system. We must bear in mind that any wrong putative TIS may end in a false gene being recognized.

**Fig. 2:**
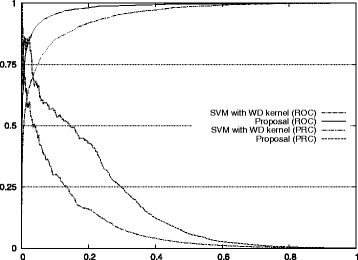
ROC/PRC curves for TIS prediction for chromosome 1. The figure shows the ROC/PRC curves for TIS prediction for chromosome 1

**Fig. 3:**
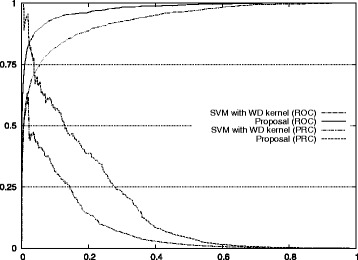
ROC/PRC curves for TIS prediction for chromosome 3. The figure shows the ROC/PRC curves for TIS prediction for chromosome 3

**Fig. 4:**
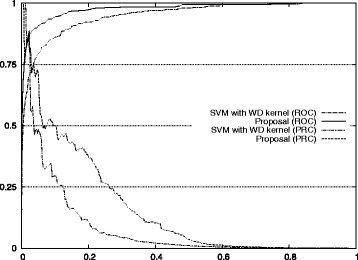
ROC/PRC curves for TIS prediction for chromosome 13. The figure shows the ROC/PRC curves for TIS prediction for chromosome 13

**Fig. 5:**
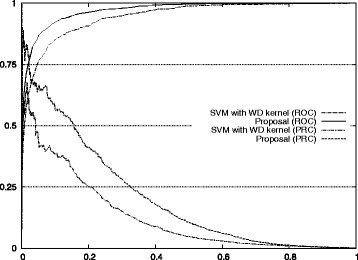
ROC/PRC curves for TIS prediction for chromosome 19. The figure shows the ROC/PRC curves for TIS prediction for chromosome 19

**Fig. 6:**
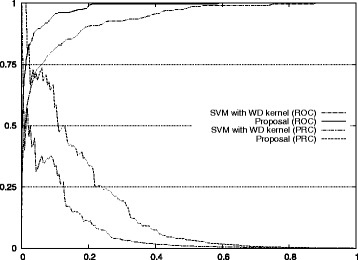
ROC/PRC curves for TIS prediction for chromosome 21. The figure shows the ROC/PRC curves for TIS prediction for chromosome 21

Figure 1 also shows the improvement of our approach with respect to the standard method for auROC and auPRC measures.^2^ Figures 2, 3, 4, 5, and 6 display the ROC and PRC curves for all the described datasets. The figures show that our approach obtained a better shaped curve in all cases and for both measures. This is interesting as it means that regardless of the classification threshold set our method would always beat the standard approach.

### Results for stop codon recognition

As previously stated we also addressed the problem of stop codon prediction. From the point of view of performance considerations stop codon recognition is a harder problem than TIS prediction. For TIS prediction we have only a codon to consider, for STOP codon three different codons could be a stop codon, thus the number of putative stop codons is multiplied by three, making the problem more imbalanced and difficult. As an example, the best current method found more than six million false positive stop codons for the five tested human chromosomes. Any program for gene recognition would be very negatively affected by such huge number of wrong putative stop codons. However, this also means that the possibilities for improving the performance of the current methods for this problem are higher.

As stated, one of the advantages of our approach is that we can optimize for the performance measure we are interested in, whether it is *G*-mean, auROC, auPRC or any other useful metric. Thus, as for TIS recognition, we carried out experiments using three performance measures: *G*-mean, auROC and auPRC. Again we found that the combination of best models obtained for each measure was different. In fact, more variation was found for stop codons than for TIS recognition.

For each of the five studied chromosomes, we obtained three different combinations of models, each one aiming at optimization of one of the three measures mentioned above. Table 6 shows the models selected for the best combination for each measure and each chromosome. As it did for TIS recognition, the constructive method always outperformed the destructive method. The latter always obtained combinations of more models that yielded to over-fitting and worse performance. It is also interesting to note the homogeneous behavior across the different chromosomes. For all five chromosomes, the combination that achieved the best results was the sum for auROC and auPRC and the majority for *G*-mean. There was only one exception, the best combination method for *G*-mean for chromosome 13 was the maximum. However, the combination based on the maximum output was the best-performing method just for this one case. In this latter combination method, the effect of a bad classifier was too harmful to obtain good performance. In this paper, for brevity’s sake, only the best models are reported.

**Table 6:** The table shows the models selected for all methods and the five studied chromosomes for stop codon recognition

			CE					AC					DM					OA					HS					OC					BT				
Chrom.	Obj.	#	S	C	K	P	W	S	C	K	P	W	S	C	K	P	W	S	C	K	P	W	S	C	K	P	W	S	C	K	P	W	S	C	K	P	W
1	auROC	4																																			
	auPRC	22																																X	X		
	G	2																																			
3	auROC	4																																			
	auPRC	10																		X										X					X		
	G	4	X																																		
13	auROC	5																																			
	auPRC	20																		X															X		
	G	6																																			X
19	auROC	5																																			
	auPRC	15																		X					X					X					X		
	G	2																																			
21	auROC	4																																			
	auPRC	18																		X																	
	G	2																																			
			CJ					FA					EC					CLF					GG					MD					SM				
Chrom.	Obj.	#	S	C	K	P	W	S	C	K	P	W	S	C	K	P	W	S	C	K	P	W	S	C	K	P	W	S	C	K	P	W	S	C	K	P	W
1	auROC	4																		X																	
	auPRC	22		X											X															X							
	G	2																																			
3	auROC	4																		X																	
	auPRC	10																																			
	G	4					X										X																				
13	auROC	5					X								X					X																	
	auPRC	20		X										X	X							X								X							
	G	6					X																														
19	auROC	5					X								X																						
	auPRC	15		X	X										X																						
	G	2																																			
21	auROC	4													X																						
	auPRC	18											X																								
	G	2																																			
0			MM					DR					MaM					TR					PT					SS					RN				
Chrom.	Obj.	#	S	C	K	P	W	S	C	K	P	W	S	C	K	P	W	S	C	K	P	W	S	C	K	P	W	S	C	K	P	W	S	C	K	P	W
1	auROC	4															X										X										
	auPRC	22					X							X	X		X			X		X		X						X			X				
	G	2															X										X										
3	auROC	4															X										X										
	auPRC	10					X								X					X					X												
	G	4															X																				
13	auROC	5															X										X										
	auPRC	20			X		X							X	X							X		X						X							
	G	6															X										X										
19	auROC	5															X										X										
	auPRC	15			X		X															X															
	G	2															X										X										
21	auROC	4															X										X										
	auPRC	18			X		X							X								X		X	X												
	G	2															X										X										
			BT.RNA					DR.RNA					HS.RNA					MM.RNA					RN.RNA					SS.RNA					XT.RNA				
Chrom.	Obj.	#	S	C	K	P	W	S	C	K	P	W	S	C	K	P	W	S	C	K	P	W	S	C	K	P	W	S	C	K	P	W	S	C	K	P	W
1	auROC	4																										X									
	auPRC	22			X							X							X	X								X		X			X		X		
	G	2																																			
3	auROC	4																										X									
	auPRC	10																										X		X			X				
	G	4																																			
13	auROC	5																										X									
	auPRC	20								X		X						X										X					X				
	G	6																															X				
19	auROC	5																															X				
	auPRC	15			X							X								X								X		X			X				
	G	2																																			
21	auROC	4																										X									
	auPRC	18		X	X					X		X							X						X				X	X			X		X		
	G	2																																			

S stands for stop codon method, C for C4.5, K for *k*-NN, P for PWM and W for a SVM with a WD string kernel

Regardless of the optimized measure, there were a few species that never appeared in the best combination: AC, DM, FA, GG, SM, and DR. As was the case for TIS recognition, although the contributions of certain species were more relevant than others, the information from many genomes was useful for the prediction of human stop codons, even those species with a large distance from the human genome. It is interesting to note that classifiers trained on the human genome were used just once and for mRNA HS sequences only four times. The analysis of the behavior showed that the information found in the human genome was redundant after a few other species were added and then its inclusion did not improve the overall performance.

For the three different measures, auROC, auPRC and *G*-mean, the obtained combinations of models are quite different. That means that we must consider which our aim before designing our classifier. This same behavior was observed for TIS recognition. However, here the situation is less stable, with more variations among chromosomes.

Regarding the classification models, PWM was never chosen. The stop codon method was chosen for several species, specially for mRNA sequences. The decision tree trained with the C4.5 algorithm was selected several times, but the *k*-NN rule and the SVM method with a string kernel were the most frequently selected methods. These results are similar to the ones obtained for TIS recognition.

With respect to the three different objectives, optimizing the *G*-mean showed the most stable results. For the five chromosomes, the SVM method for MaM and PT was always selected, with the exception of chromosome 3. However, additional models were selected for each chromosome that varied from one to annother. Surprisingly, CE was selected for chromosome 3, despite its large evolutionary distance to human. This result supports the idea that selecting the genomes in an intuitive way is not optimal. For auROC, four or five models were always selected, although not the same models for every chromosome. The SVM method for MaM and PT was always chosen, but the remaining methods depended on the chromosome. This is another interesting result because most stop codon recognition programs rely on common models for any task. Finally, for auPRC, significantly more models were selected, from 10 to 22, with a significant variation between the chromosomes.

**Fig. 7:**
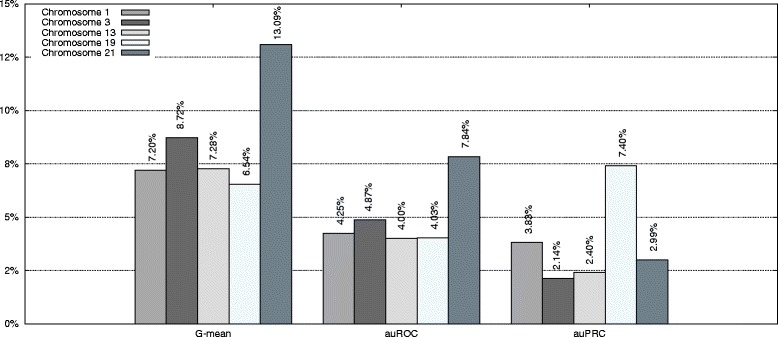
Improvement for stop codon recognition. The figure shows the improvement of our approach with respect to the standard approach of using the best performing classifier

The next step was to compare the performances of our approach and the standard method of choosing the best performing classifier. A summary of the results for stop codon recognition of the five studies chromosomes is shown in Table 7. The first interesting result is that the proposed approach beat the standard approach for all measures and all chromosomes. The improvements in auROC, auPRC and *G*-mean are shown in Fig. 7. Again, our approach was reasonably fast, in the worst case 9702 seconds were needed to obtain the best combination.

**Table 7:** The table shows the specificity (Sp), sensitivity (Sn), true positives (TP), true negatives (TN), false negatives (FN), false positives (FP) and area under the ROC and PRC curves (auROC/PRC) as well as the time needed for obtaining the best combination for all methods and the five studied chromosomes for stop codon recognition

Chromosome	Objective	Method	Combination	auROC	auPRC	G	Sp	Sn	TP	FN	TN	FP	#models	Time(s)
Chr. 1	-	Std	-	0.9280	0.0142	0.8487	0.8942	0.8055	1735	419	21,077,932	2,495,099	-	-
	auROC	Cons	Sum	**0.9705**	0.0368	0.8868	0.9707	0.8101	1745	409	22,882,052	690,979	4	4818
	auPRC	Cons	Sum	0.9522	**0.0525**	0.8165	0.9762	0.6829	1471	683	23,011,801	561,230	22	9702
	G	Cons	Majority	0.9307	0.0013	**0.9207**	0.9426	0.8993	1937	217	22,218,892	1,354,139	2	1223
Chr. 3	-	Std	-	0.9233	0.0083	0.8256	0.9159	0.7442	829	285	19,711,889	1,810,611	-	-
	auROC	Cons	Sum	**0.9720**	0.0262	0.8959	0.9676	0.8294	924	190	20,826,101	696,399	4	4,094
	auPRC	Cons	Sum	0.9421	**0.0297**	0.7810	0.9735	0.6266	698	416	20,951,286	571,214	10	8019
	G	Cons	Majority	0.9584	0.0015	**0.9128**	0.9462	0.8806	981	133	20,364,397	1,158,103	4	1719
Chr. 13	-	Std	-	0.9185	0.0071	0.8150	0.9103	0.7297	243	90	9,902,079	976,223	-	-
	auROC	Cons	Sum	**0.9585**	0.0156	0.8817	0.9733	0.7988	266	67	10,587,495	290,807	5	4542
	auPRC	Cons	Sum	0.9392	**0.0311**	0.7604	0.9824	0.5886	196	137	10,687,114	191,188	20	9136
	G	Cons	Majority	0.9502	0.0106	**0.8878**	0.9545	0.8258	275	58	10,383,296	495,006	6	1619
Chr. 19	-	Std	-	0.9328	0.0379	0.8515	0.8664	0.8368	1190	232	4,042,574	623,230	-	-
	auROC	Cons	Sum	**0.9731**	0.0843	0.9160	0.9335	0.8987	1278	144	4,355,655	310,149	5	3878
	auPRC	Cons	Sum	0.9557	**0.1119**	0.8787	0.9250	0.8347	1187	235	4,316,077	349,727	15	8806
	G	Cons	Majority	0.9346	0.0026	**0.9169**	0.9002	0.9339	1328	94	4,199,984	465,820	2	856
Chr. 21	-	Std	-	0.8890	0.0083	0.7778	0.9191	0.6582	156	81	3,425,375	301,584	-	
	auROC	Cons	Sum	**0.9674**	0.0383	0.8797	0.9654	0.8017	190	47	3,597,983	128,976	4	2379
	auPRC	Cons	Sum	0.9455	**0.0382**	0.7970	0.9713	0.6540	155	82	3,620,079	106,880	18	8134
	G	Cons	Majority	0.9199	0.0007	**0.9087**	0.9320	0.8861	210	27	3,473,463	253,496	2	507

The results of our method for the three different evaluation measures are shown in boldface

The differences were significant. For *G*-mean, in the worst case, the improvement was 6.54 %, and in the best case, it was 13.09 %. For auPPRC, the results showed an improvement from 2.14 to 7.40 %. For auROC, the improvement was also significant, ranging from 4.00 to 7.84 %.

Table 8 shows the relative improvement of our approach in terms of true positives, false negatives, true negatives and false positives. From the table, we can see how our approach was able to improve the false negative results in the worst case by 35 % and in the best case by 66 %. This is a relevant reduction, as many of the current gene recognition programs rely on the classification of stop codons; therefore, it is very likely that the genes whose stop codon is not correctly predicted would be missed by the gene recognizer or at least wrongly predicted.

**Table 8:** Relative improvement for true positives, false negatives, true negatives and false positive of our approach over the best method for stop codon recognition

Chromosome	True	False	True	False
	positive	negative	negative	positive
Chr.1	11.64 %	48.21 %	5.41 %	45.73 %
Chr. 3	18.34 %	53.33 %	3.31 %	36.04 %
Chr. 13	13.17 %	35.56 %	4.86 %	49.29 %
Chr. 19	11.60 %	59.48 %	3.89 %	25.26 %
Chr. 21	34.62 %	66.67 %	1.40 %	15.95 %

Furthermore, our method was also able to improve the true negative rate, from 1 to 5 % depending on the chromosome. Therefore, any annotation system that uses our approach would have a significantly reduced set of putative TISs and better expected performance. This is especially true when a large amount of false positives is found the by the standard approach, which is an actual problem for any automatic annotation system.

**Fig. 8:**
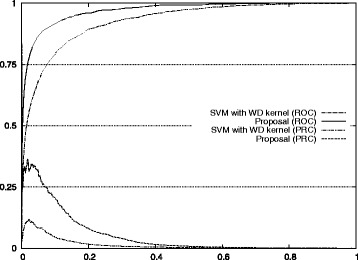
ROC/PRC curves for stop codon prediction for chromosome 1. The figure shows the ROC/PRC curves for stop codon prediction for chromosome 1

**Fig. 9:**
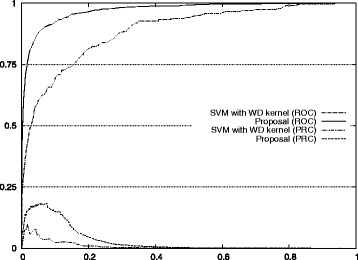
ROC/PRC curves for stop codon prediction for chromosome 3. The figure shows the ROC/PRC curves for stop codon prediction for chromosome 3

**Fig. 10:**
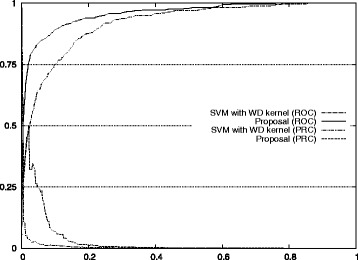
ROC/PRC curves for stop codon prediction for chromosome 13. The figure shows the ROC/PRC curves for stop codon prediction for chromosome 13

**Fig. 11:**
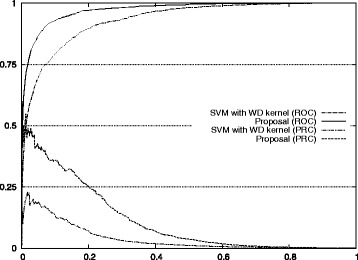
ROC/PRC curves for stop codon prediction for chromosome 19. The figure shows the ROC/PRC curves for stop codon prediction for chromosome 19

**Fig. 12:**
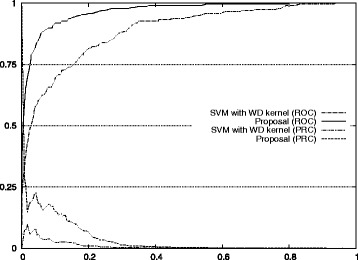
ROC/PRC curves for stop codon prediction for chromosome 21. The figure shows the ROC/PRC curves for stop codon prediction for chromosome 21

The improvements for auROC and auPRC values are also shown in Fig. 7. The actual ROC and PRC curves are shown in Figs. 8, 9, 10, 11 and 12. These figures show that our approach improved the auROC and auPRC for all five studied chromosomes. These results demonstrate that the overall performance of the proposed method was better than the performance of best model. The actual ROC and PRC curves shown in Figs. 8, 9, 10, 11 and 12 show that the curves corresponding to our proposal are always above the curves of the best model. This indicates better performance for all the possible thresholds of classification.

Figure 7 also shows the improvement of our approach with respect to the standard method for auROC and auPRC measures. Figures 8, 9, 10, 11, and 12 display the ROC and PRC curves for all the described datasets. The figures show that our approach obtained a better shaped curve in all cases and for both measures. This is interesting as it means that regardless of the classification threshold set our method would always beat the standard approach and that indicates improved performance for any given threshold.

As a final comparison, we performed a Wilcoxon test to compare the results of our approach as the best current method for both TIS and stop codon prediction. we used the Wilcoxon test for comparing pairs of algorithms. We chose this test because it assumes limited commensurability and is safer than parametric tests, because it does not assume normal distributions or homogeneity of variance. Furthermore, empirical results [21] show that this test is also stronger than other tests. The *p*-value of the test was of 0.005062 for the three evaluation measures, auROC, auPRC and *G*-mean. This means that our approach beat the standard one at a confidence level of 99 %.

## Conclusions

In this paper, we presented a new approach for functional site recognition in genomic sequences. The approach consists of a stepwise procedure that can combine tens or hundreds of classifiers trained on different sequences and using genomic information from different species. The approach is rapid and can be used for the recognition of any type of functional site. Our method substitutes the current approach of selecting the species to be used heuristically based on biological considerations. Our results have proven that that methodology is suboptimal because species that are not considered in previous works have been shown useful in our experiments.

Although we have focused our experiments on the case of the combination of multiple species, we can also use the proposed approach for combining classifiers trained on different sequences of the same species, or classifiers trained using different parameters or learning procedures.

Furthermore, with our method, we can optimize any measure we are interested in. For instance, in the reported experiments, we have shown how we can focus on the optimization of *G*-mean, auROC or auPRC measures. The results have shown that the combination of classifiers that optimizes each one of these measures can be very different, supporting our separate approach.

To provide the necessary focus, we restrict the experimental study of our method to TIS and stop codon recognition. The reported results show that the proposed method exhibits improved sensitivity, specificity, auROC and auPRC compared with the standard approach of using the best available classifier. The results show a remarkable improvement in the *G*-mean, auROC and auPRC measures. Most of the best state-of-the-art gene prediction systems use a first step of functional site recognition, thus as the proposed method significantly improves this site recognizers it has the potential for improving any annotation system.

## Availability and supporting data

The data sets supporting the results of this article are available at http://cib.uco.es/index.php/supplementary-material-for-stepwise-site-prediction. The source code, in C and licensed under the GNU General Public License, used for all methods is also available in the same link. The code only uses GPL libraries and so it should be able to compile in any system. SVMs were programmed using the LIBSVM library [22].

## Endnotes

^1^The acronyms in parentheses will be used across the paper to refer to the corresponding species.

^2^The experiment were always carried out using all the negative samples for evaluating the classification performance. For the worst case the ratio minority/majority class is almost 1:11000, thus low auPRC values are obtained by any method. Only a few thousands FPs among several millions of TNs would obtain a very low precision value. The results for stop codon recognition are worse due to a larger number of TNs sequences.
